# Examination of Relationships Between 24-Hour Movement Behaviors and Mental Health Outcomes in Frontline Workers: Protocol for a Scoping Review

**DOI:** 10.2196/90813

**Published:** 2026-05-27

**Authors:** Amalie Skovgaard, Alison Kirk, Nicola Cogan

**Affiliations:** 1 Department of Psychological Sciences and Health, University of Strathclyde, 40 George Street, Glasgow, G1 1QE, United Kingdom, 44 0141 552 4400; 2 Psychological Services, NHS Lanarkshire, Wishaw, United Kingdom

**Keywords:** frontline workers, mental health, psychophysiology, 24-hour movement, sleep, physical activity, occupational health

## Abstract

**Background:**

Frontline workers across multiple occupations operate in high-stress, trauma-exposed environments characterized by chronic demands and irregular schedules, increasing risk of burnout, depression, and poor sleep. Emerging evidence highlights the role of 24-hour movement behaviors in relation to mental health. Despite growing attention, research remains fragmented and often focuses on individual behaviors rather than their combined influence. This protocol outlines a scoping review designed to map existing evidence and identify research gaps.

**Objective:**

The primary aim is to map research examining relationships between 24-hour movement behaviors and mental health outcomes in frontline workers. Objectives include examining measurement approaches, associations, and methodological gaps.

**Methods:**

This scoping review will follow the JBI methodology and the PRISMA-ScR (Preferred Reporting Items for Systematic Reviews and Meta-Analyses extension for Scoping Reviews) guidelines. Eligible studies include English-language research published between 2000 and 2025 involving adult frontline workers across multiple occupations and examining movement behaviors and mental health outcomes. A three-step search strategy will be conducted across multiple databases alongside targeted gray literature searches. Screening and data extraction will be performed independently by two reviewers. Findings will be synthesized using tabular summaries and narrative synthesis.

**Results:**

Preliminary searches and pilot-testing were completed in October 2025. As of May 2026, updated database and supplementary searches have been completed, with 527 studies meeting the inclusion criteria for data charting. Data charting and synthesis are currently underway. Completion of the review and submission for publication are anticipated in July 2026.

**Conclusions:**

This review will provide a comprehensive overview of research linking 24-hour movement behaviors and mental health in frontline workers. Findings will highlight methodological, occupational, and research gaps to inform future research, policy, and practice.

## Introduction

### Background

Frontline workers are individuals whose roles require direct engagement with the public during routine and critical operations, often in unpredictable or high-stakes environments [1,2]. Although much of the empirical literature has historically focused on health care staff, broader definitions increasingly recognize that emergency responders, firefighters, police officers, transport personnel, and other essential workers also perform frontline duties that are psychologically and operationally demanding [3,4]. Across these sectors, workers operate within environments characterized by chronic stress exposure, trauma, moral responsibility, and irregular schedules, yet their contribution is fundamental to societal functioning, safety, and public health resilience [3,5,6]. Frontline workers are 23%‐39% more likely to experience clinically significant depression and 27%‐40% more likely to experience anxiety than the general population [7], with meta-analytic evidence confirming a higher prevalence of depression (31% vs 25%) and anxiety (31% vs 27%) among health care staff [6,8]. Qualitative findings further suggest that distress is often underreported due to professional expectations [9], emphasizing the need for proactive strategies to support psychological sustainability.

### Linking Movement Behaviors and Stress Indicators

Growing attention is being directed toward 24-hour movement behaviors (24hrMBs), defined as the integrated pattern of sleep, sedentary time, light activity, and moderate-to-vigorous physical activity. These behaviors influence both physiological and mental health [10,11]. While higher levels of physical activity are often associated with improved mental health, occupational physical activity in manual or physically demanding roles may contribute to long-term musculoskeletal strain or fatigue, indicating that the relationship between activity levels and well-being may be more complex in certain frontline occupations [10]. Disruptions in this pattern, such as insufficient sleep or prolonged sedentary time, have been linked to heightened stress, worsened mood, and reduced well-being [12]. Frontline work presents additional challenges, including extended working hours, night shifts, rotating schedules, acute trauma exposure, and circadian misalignment (a mismatch between an individual’s internal biological rhythms and externally imposed work schedules), which can disrupt rest-activity cycles and amplify stress responses [13-15].

Complementing behavioral evidence, psychophysiological markers are objective indicators of autonomic nervous system activity and physiological stress regulation. Common measures include heart rate, heart rate variability (HRV), galvanic skin response, respiratory rate, and actigraphy-derived sleep indices, which can provide measurable indicators of stress, recovery, and autonomic regulation [16,17]. For instance, lower HRV has been consistently associated with burnout and anxiety in health care and emergency workers [18,19]. Advances in wearable and sensor-based technologies now allow continuous, ecologically valid monitoring of these markers, offering opportunities to integrate physiological and behavioral data to better understand occupational stress responses [19-21]. Psychophysiological markers captured through wearable or sensor-based technologies may reflect both acute stress responses and longer-term physiological dysregulation. For example, short-term changes in HRV, electrodermal activity/galvanic skin response, or respiratory rate may indicate immediate autonomic responses to stressors, whereas sustained alterations in resting HRV or sleep fragmentation may reflect chronic occupational strain or burnout [16,18,19].

Given the high prevalence of mental health difficulties, including burnout, posttraumatic stress disorder symptoms, emotional dysregulation, and moral injury among frontline workers [1,22], understanding how behavioral and physiological indicators interact may be critical for early detection, monitoring, and intervention. Such integration aligns with global public health priorities, including the World Health Organization’s 24-hour movement guidelines and occupational well-being frameworks, emphasizing the need for multidimensional and data-informed prevention strategies [11,23]. Despite this, research on 24hrMBs and mental health in frontline populations remains fragmented [24]. Most studies examine single behaviors (eg, sleep, exercise) rather than their interrelationships, and methodological approaches vary widely, including differences in device types, sampling protocols, and measurement techniques. Compositional data, which capture the interdependent nature of these behaviors, are rarely used, and contextual factors and adherence are inconsistently reported [24]. Similarly, psychophysiological and psychological measures of occupational stress are seldom integrated within a single analytic model, limiting understanding of how physiological dysregulation manifests behaviorally and psychologically [17,21]. Research often focuses on isolated sectors, primarily health care, underrepresenting policing, firefighting, and emergency medical services, restricting a holistic understanding of shared and unique stressors across frontline professions [3,4].

### Current Gap

The well-being of frontline workers is increasingly recognized as a strategic priority, with the World Health Organization [5,23] advocating proactive, data-informed occupational health frameworks. Advances in wearable and sensor technologies now allow simultaneous monitoring of behavioral and physiological indicators; however, integration of 24hrMB compositions with mental health outcomes remains limited [17,20,24]. Without systematically combining these measures, the evidence base remains fragmented, hindering early identification of stress, development of proactive interventions, and establishment of integrated frameworks to enhance workforce resilience. A scoping review is therefore well-suited to map this heterogeneous evidence base. By systematically charting studies examining 24-hour movement behaviors alongside mental health indicators in frontline workers, this review will identify common measure combinations, highlight underrepresented sectors, and inform the development of integrated assessment frameworks. Ultimately, findings may guide future research, support targeted interventions, and facilitate the translation of wearable and movement-based monitoring into occupational health policy, contributing to sustainable workforce resilience.

### Scoping Review Questions

This review aims to map research examining relationships between 24hrMBs and mental health outcomes in frontline workers, while also identifying methodological approaches used in the literature.

The primary question the review aims to answer is as follows:

1. What is the current state of evidence examining the relationships between 24-hour movement behaviors and mental health outcomes among adult frontline workers?

The secondary questions are as follows:

1. How are 24hrMBs defined and assessed across frontline populations?
2. What associations have been reported between movement behaviors and mental health outcomes in frontline workers?
3. What study designs, measurement tools, and contexts are represented in the literature, and where do research gaps exist?
4. How have movement behaviors and mental health outcomes been examined across different study focuses and contexts in frontline worker research, and what implications may this have for policy or practice?

## Methods

This scoping review aims to systematically map the current literature, elucidating key characteristics, methodologies, findings, and interventions related to 24hrMBs, mental health, and frontline workers, while identifying knowledge gaps to inform future research. The review will be conducted in accordance with the methodological framework and guidelines established by the JBI for scoping reviews and will be reported following the PRISMA-ScR (Preferred Reporting Items for Systematic Reviews and Meta-Analyses extension for Scoping Reviews) guidelines [25,26].

### Ethical Considerations

As this scoping review uses only existing, publicly accessible literature and does not involve the collection of new data from human participants, formal ethical approval is not required. Nevertheless, the review will be conducted with careful attention to ethical standards for secondary research, including accurate representation of findings, proper attribution of all original sources, and transparent reporting of methods in line with PRISMA-ScR recommendations [26].

### Eligibility Criteria

#### Population

For the purposes of this review, “frontline workers” are defined as adults aged 18 years and older employed in civilian occupations characterized by high levels of stress exposure, public-facing responsibilities, or frequent exposure to emergency or traumatic situations. This includes health care personnel, emergency responders, firefighters, and law enforcement personnel. No upper age limit will be imposed to allow for the inclusion of all adult workers. Participants of any nationality will be eligible provided they hold relevant frontline roles. Studies focusing exclusively on military personnel, individuals under 18 years of age, clinical populations without frontline occupational exposure, or nonhuman subjects will be excluded. Military populations are excluded because their operational environments, organizational structures, training regimes, and deployment contexts differ substantially from civilian frontline occupations [3,27]. These differences may involve unique exposure profiles and stress mechanisms that fall outside the scope of the present review.

Mixed samples including military or clinical subgroups will be included only if data specific to civilian frontline workers can be separately extracted; otherwise, they will be documented as mixed samples but excluded from synthesis. Given the diversity of frontline occupations, studies will also be categorized by occupational group during synthesis (eg, health care professionals, emergency service personnel, and other frontline roles) to allow comparison across sectors and reduce the risk of overgeneralizing findings across occupational contexts with differing stressors and work demands [28,29].

#### Concept

This review will include studies that explicitly examine at least one 24hrMB in relation to at least one mental health or trauma-related outcome among adult civilian frontline workers. For the purposes of this review, 24hrMBs are defined as sleep, sedentary behavior, light physical activity, moderate-to-vigorous physical activity, or combinations of these behaviors across a 24-hour period. To reduce conceptual heterogeneity, studies will be included only where the movement behavior is measured or described as a substantive exposure, outcome, correlate, intervention target, or monitoring variable, rather than being mentioned only as background context. Mental health outcomes will be organized into predefined domains during charting: stress, burnout, depression, anxiety, posttraumatic stress or trauma-related symptoms, and well-being. Studies using alternative terminology, such as occupational strain, moral injury, compassion fatigue, or subjective experiences of stress and recovery, will be eligible only where these constructs clearly and directly correspond to one of the predefined domains and are examined alongside at least one movement behavior. During synthesis, findings will be grouped by both movement behavior domain and mental health outcome domain to support a structured interpretation of a heterogeneous evidence base. Where classification is unclear, studies will be discussed among reviewers to ensure consistent alignment with predefined domains.

#### Context

The review will focus on research conducted in occupational environments where frontline or high-stress work occurs, including health care, emergency services, firefighting, and policing. Studies conducted in clinical, workplace, field, or experimental settings that collect data on movement behaviors will be considered. Comparative studies examining different frontline professions, geographic locations, or countries will also be included where they provide insight into occupational stress, trauma exposure, and 24hrMBs across diverse frontline contexts.

#### Date of Publication

Studies published between 2000 and 2025 will be eligible, reflecting a contemporary period of research. This time frame was selected to capture the emergence and increasing adoption of wearable and digital health monitoring technologies in the early 2000s, alongside evolving research on occupational stress and mental health among frontline workers [30,31].

#### Type of Evidence Source

This review will include empirical primary research using quantitative, qualitative, or mixed methods designs, including observational, interventional, experimental, and case study designs where they report original empirical data relevant to the population, concept, and context. Evidence syntheses, including systematic reviews and scoping reviews, will be excluded to avoid duplication of primary data and to ensure that the unit of analysis remains consistent across included studies. Gray literature, such as organizational reports, policy guidance, professional body reports, or technical white papers, will be included only where it provides substantive empirical or practice-based information on workplace practices, interventions, monitoring technologies, or implementation approaches relevant to 24-hour movement behaviors and mental health in frontline workers. Gray literature will be mapped descriptively and will not duplicate findings already represented in peer-reviewed primary studies. Nonscholarly sources, such as blogs, news articles, and opinion pieces lacking empirical or policy-relevant data, will be excluded. This approach ensures a focused and methodologically consistent evidence base while maintaining breadth in mapping applied and practice-oriented research. Evidence syntheses (eg, systematic and scoping reviews) may be referenced to contextualize the background and rationale of the review but will not be included as sources of evidence in the synthesis.

#### Language

Only studies published in English will be included to maintain consistency in assessing methodological quality, accurately extracting data, and interpreting technical terminology related to 24hrMBs and mental health. Including non-English studies could introduce translation challenges that might affect the accuracy and reliability of the review findings.

### Search Strategy

A structured three-step search strategy will be implemented following the JBI methodology for scoping reviews. An initial exploratory search was carried out via EBSCOhost using APA PsycInfo and CINAHL (full text) to identify appropriate keywords, subject headings, and search string combinations related to 24hrMBs and mental health outcomes among frontline workers. These databases were selected for their comprehensive coverage of psychology, behavioral science, physiology, occupational health, and trauma research. Insights from this preliminary search informed the refinement of search terms and the development of concept groupings encompassing movement, physiological monitoring, mental health, and frontline occupational roles. Building on this initial work, the search will be expanded to include APA PsycInfo, CINAHL, MEDLINE, SPORTDiscus, PubMed, and Scopus, ensuring coverage across health sciences as well as behavioral, occupational, and psychophysiological domains relevant to frontline workers. Gray literature will also be searched, including organizational reports (eg, NHS Scotland workforce well-being reports), policy briefs and guidance (eg, GOV.UK Wellbeing and mental health: Applying All Our Health), and technical white papers from research institutes or professional organizations (eg, British Psychological Society reports on resilience in emergency service personnel). Search terms were developed in consultation with an academic librarian and refined according to JBI guidance [32]. Draft strategies for APA PsycInfo and MEDLINE are provided in Multimedia Appendix 1, with adaptations for the other databases included in supplementary materials in the final manuscript. All retrieved references will be managed using EndNote [33] reference management software and imported into Rayyan [34], a platform designed to support collaborative article screening and study selection.

### Sources of Evidence

Before commencing the screening process, a calibration exercise will be conducted to ensure consistent application of inclusion criteria among reviewers [35]. Screening will follow a three-stage process: (1) title review, (2) abstract review, and (3) full-text review. Separating title and abstract screening allows rapid removal of clearly irrelevant records in large search outputs while maintaining inclusion sensitivity. Records identified as potentially relevant by at least one reviewer will progress to the subsequent screening stage. Disagreements at this stage will be resolved using an inclusion-favoring approach, in line with JBI guidance. During full-text review, two reviewers will independently assess each study against predetermined inclusion criteria encompassing population, concept, context, evidence type, and publication date. Any disagreements will be resolved through discussion, and where consensus cannot be reached, a third reviewer will adjudicate. Rayyan will be used to organize citations, document reasons for exclusion, and manage the review workflow. The final study selection process will be summarized using a PRISMA-ScR flow diagram illustrating the number of records identified, screened, excluded, and included in the review [26]. This structured approach will support methodological rigor and transparency in identifying evidence at the intersection of 24hrMBs and mental health among frontline workers [36].

### Stakeholder Consultation

Stakeholder consultation will be incorporated at multiple stages of the review to ensure the relevance and applicability of findings. Approximately 5‐10 stakeholders will be consulted, including health care professionals, occupational health researchers, and representatives from organizations supporting frontline worker well-being. Stakeholders will be identified through professional networks, research collaborators, and relevant organizational contacts with expertise in frontline occupational health or mental well-being. Consultation will occur at key stages of the review process. Early consultation will support refinement of the research questions and eligibility criteria. A second consultation stage will occur following initial data charting to gather feedback on emerging themes, the interpretation of findings, and the identification of potential gaps in the literature. Consultation will be conducted through structured meetings and email correspondence. This participatory approach aligns with the principles of co-development and supports methodological transparency, ensuring the review reflects the priorities and experiences of frontline populations [37].

### Data Extraction

Data extraction will be conducted using a standardized charting form developed in accordance with JBI guidance and tailored to the review’s research questions. The form will be piloted independently by three reviewers on a sample of 5 studies to assess clarity, completeness, and relevance. Any inconsistencies or ambiguities identified during piloting will be discussed, and the form will be refined before full data extraction. Variables extracted from included studies will be coded where appropriate to support structured synthesis across studies. Coding refers to the process of assigning extracted information to predefined analytical categories based on the data charting framework presented in Table 1, consistent with established scoping review methodology [25,38]. This approach allows information reported across studies to be systematically organized and compared during synthesis. Where study information does not clearly fit predefined categories, additional categories may be created inductively during the charting process to ensure accurate representation of the literature. For example, where studies include participants classified as “other frontline workers,” the specific occupational roles will be examined in greater detail and, where appropriate, further subcategorized during synthesis. Consistent with scoping review methodology, formal appraisal or categorization of the technical validity of wearable monitoring devices will not be undertaken [38]. Instead, information regarding the movement measurement method and monitoring devices used in each study will be extracted to provide contextual insight into the monitoring technologies represented across the literature.

**Table 1.** Data extraction framework.

Variable	Description (coded where appropriate)	Example
Author and year	First author and publication year	Smith 2021
Country/region	Country where the study was conducted	United States
Population	Type of frontline worker studied (recorded as reported and coded where appropriate into occupational groups such as health care, emergency services, law enforcement, firefighters, mixed frontline workers, or other frontline occupations)	Health care workers
Study design	Overall research design (coded as cross-sectional, longitudinal, interventional, experimental, qualitative, or mixed methods)	Cross-sectional
Movement behavior(s)	Behavior(s) examined in the study (coded as physical activity, sleep, sedentary behavior, or multiple behaviors)	Sleep
Movement measurement method	Method used to assess movement behaviors (coded as self-report, accelerometer, actigraphy, wearable devices, or mixed methods)	Self-report
Monitoring device/sensor	Device used to collect behavioral or physiological data	Actigraphy watch
Mental health outcome	Mental health outcome(s) examined (coded as stress, burnout, depression, anxiety, posttraumatic stress disorder/trauma, well-being, or multiple outcomes)	Burnout
Study focus	Type of relationship examined (coded as association study, monitoring study, or intervention study)	Association study
Study context	Context in which the study occurred (coded as routine conditions, pandemic, war/conflict, natural disaster, another crisis, or unclear)	COVID-19

Data will be synthesized descriptively using a narrative approach. Quantitative findings, such as physiological metrics and activity levels, will be summarized using tables and descriptive statistics, while qualitative data will undergo thematic analysis to identify patterns related to occupational, contextual, and experiential aspects of stress and well-being in frontline workers. The synthesis will map relationships between movement behaviors and mental health outcomes, while highlighting methodological diversity and gaps in current research. Data extraction will be conducted by three reviewers. Discrepancies will be resolved through discussion, with a fourth reviewer consulted where necessary to maintain accuracy, consistency, and reliability throughout the charting process.

## Results

### Search and Selection Progress

**Figure 1.**
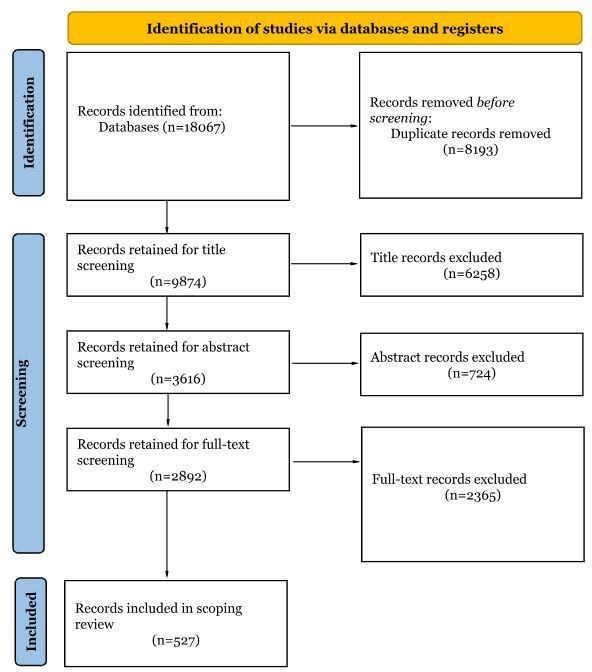
Updated PRISMA-ScR flow diagram for study identification, screening, eligibility assessment, and inclusion. PRISMA-ScR: Preferred Reporting Items for Systematic Reviews and Meta-Analyses extension for Scoping Reviews.

Preliminary exploratory searches were conducted in October 2025 to identify relevant keywords, subject headings, and search string combinations related to 24hrMBs and mental health outcomes in frontline workers. Pilot-testing of the eligibility criteria was undertaken using a sample of studies to ensure consistent application of inclusion criteria and calibration among reviewers. Insights from this process informed refinement of the search terms and development of the final search strategy described in the Methods section. As of May 2026, updated database and supplementary searches have been completed. A total of 9874 records were identified and managed using EndNote and Rayyan. Following title screening, 3616 records were retained for abstract screening, of which 2892 progressed to full-text assessment. After full-text screening, 527 studies met the inclusion criteria and have been included for data charting. These figures are presented in a PRISMA-ScR flow diagram (Figure 1). Data charting is ongoing and synthesis is underway. Completion of the review and submission for publication are expected in July 2026.

### Data Charting and Planned Synthesis

Data charting is currently underway using the structured framework described in Table 1. The planned synthesis will describe the distribution of included studies by frontline occupation, movement behavior, measurement method, mental health outcome, study design, and occupational context. Studies will also be grouped by movement behavior domain and mental health outcome domain to support structured interpretation of a heterogeneous evidence base.

## Discussion

### Strengths and Limitations

This protocol outlines a comprehensive framework for conducting a scoping review that will map the current evidence on relationships between 24hrMBs and mental health outcomes in frontline workers. The review will examine how occupational stress, trauma exposure, shift work, and job-specific demands relate to sleep, physical activity, sedentary behavior, and psychological well-being across high-stress professions, including health care, emergency services, firefighting, and policing. Previous research has often been limited in scope, focusing on specific occupational groups, isolated physiological indicators, or singular mental health outcomes [2,20]. In contrast, this review adopts a structured but inclusive approach by organizing evidence according to occupational group, movement behavior domain, mental health outcome domain, study design, evidence source type, and context. This structure is intended to preserve the breadth appropriate for a scoping review while reducing the risk that heterogeneous evidence is interpreted as a single undifferentiated body of findings. A strength of the protocol is its adherence to JBI scoping review guidance and PRISMA-ScR reporting standards, which supports transparency and reproducibility [26,32]. The search strategy is multistage and includes database searching, supplementary gray literature searching, librarian input, and pilot testing of eligibility criteria. A further strength is the explicit evidence-source plan, with empirical primary studies forming the unit of synthesis and gray literature mapped descriptively where it provides relevant empirical or practice-based information. Nevertheless, certain limitations should be acknowledged. Restricting the review to English-language publications may introduce language bias and limit global generalizability. The breadth of frontline occupations and mental health constructs may also limit comparability across studies. To address this, the synthesis will group studies by predefined domains and avoid making causal claims where study design, measurement approaches, or occupational contexts do not support them. Variations in device types, self-report measures, monitoring protocols, and definitions of movement behaviors may further limit direct comparison; therefore, findings will be interpreted descriptively using a narrative approach rather than as pooled estimates.

### Dissemination Plan

The findings from this scoping review will be shared through a combination of academic and nonacademic channels to maximize reach, stakeholder engagement, and practical impact. A peer-reviewed journal article will present the results, discussion, key findings, and conclusions, while a summary report will be prepared for organizational stakeholders, including health care providers, emergency services, professional associations, and policy bodies. Stakeholder-specific dissemination will include concise reports summarizing key findings, research gaps, and practical recommendations tailored to occupational health and workplace well-being priorities. Academic dissemination will include presentations at national and international conferences in psychology, occupational health, public health, and trauma research. Public engagement will be supported through accessible, open-access outputs, such as webinars, infographics, and short-form digital summaries, designed to communicate findings to frontline workers, practitioners, and policymakers. All outputs will be co-developed with frontline worker representatives and organizational partners to ensure relevance, accessibility, and applicability. Digital dissemination will leverage institutional websites, professional networks, and social media platforms to enhance visibility and reach. In alignment with open science principles, all review materials, including search strategies, data extraction templates, and synthesis tables, will be made available as supplementary files or will be deposited in open-access repositories. This approach promotes transparency, reproducibility, and knowledge sharing, enhancing the utility of the review for researchers, practitioners, and decision-makers across frontline sectors.

### Conclusion

In summary, this protocol establishes a timely and methodologically robust approach to understanding the relationships between 24hrMBs and mental health in frontline workers. Amid growing awareness of occupational stress, burnout, and trauma exposure, the review will address existing knowledge gaps, identify priority areas for future research, and guide the design of targeted, evidence-informed interventions to support the mental health and well-being of frontline workers.

## Supplementary material

10.2196/90813 Multimedia Appendix 1 Sample search strategy.

10.2196/90813 Checklist 1 Completed PRISMA-ScR checklist.

## Abbreviations

24hrMB: 24-hour movement behavior
HRV: heart rate variability
PRISMA-ScR: Preferred Reporting Items for Systematic Reviews and Meta-Analyses extension for Scoping Reviews
