# Supplementary material for: Examination of Relationships Between 24-Hour Movement Behaviors and Mental Health Outcomes in Frontline Workers: Protocol for a Scoping Review
Source: JMIR Res Protoc. 2026 May 27;15:e90813. doi: 10.2196/90813 (PMC13215630; doi:10.2196/90813)
Supplement: Multimedia Appendix 1 [file resprot-v15-e90813-s001.docx]

# **Sample Search Strategy for APA PsycInfo**

1. "Frontline worker*" OR "Healthcare worker*" OR "hospital staff" OR "medical staff" OR Nurse* OR Midwife* OR physician* OR doctor* OR surgeon* OR "health professional*" OR physiotherapist* OR radiographer* OR "speech therapist*" OR "psychiatric nurse*" OR "nursing assistant*" OR "healthcare assistant*" OR "residential care worker*" OR "care worker*" OR "hospice worker*" OR "support worker*" OR "Occupational stress worker*" OR "Emergency responder*" OR "First responder*" OR Paramedic* OR "Community paramedic*" OR Firefighter* OR Lifeguard* OR "Search and rescue worker*" OR "Rescue worker*" OR lifesaver* OR Police OR "police officer*" OR "law enforcement" OR "Correctional officer*" OR "prison officer*" OR "Crisis worker*" OR "Crisis hotline worker*" OR "Child protection worker*" OR "Youth worker*" OR "Substance misuse worker*" OR "Disaster relief worker*" OR "Disaster response worker*" OR "Humanitarian worker*" OR "Ambulance staff" OR "emergency medical technician*" OR EMT* OR "Emergency call handler*" OR dispatch* OR "Call handler*" OR "Critical worker*" OR "Essential worker*"
2. "24-hour movement" OR "24 hour movement" OR "24h movement" OR "movement behaviour*" OR "movement behavior*" OR "movement pattern*" OR "activity pattern*" OR "daily activity*" OR "total physical activity" OR "physical activity" OR "active behaviour*" OR "active behavior*" OR "activity level*" OR "light physical activity" OR LPA OR "moderate-to-vigorous physical activity" OR MVPA OR "sedentary behaviour" OR "sedentary behavior" OR "sedentary lifestyle" OR "sedentary time" OR "sitting time" OR "sitting behaviour*" OR "sitting behavior*" OR inactivity OR "step count*" OR "energy expenditure" OR EE OR sleep* OR SOL OR WASO OR "sleep-wake pattern*" OR "circadian rhythm*" OR rest OR napping OR "rest-activity rhythm*" OR "heart rate" OR HR OR "heart rate variability" OR HRV OR "blood pressure" OR BP OR "cardiac output" OR "electrodermal activity" OR "galvanic skin response" OR GSR OR "skin conductance" OR "skin temperature" OR "vagal tone" OR "autonomic function*" OR "autonomic nervous system measure*" OR "respiratory rate" OR "breathing rate" OR respiration OR "respiratory variability" OR actigraphy OR accelerometry OR "wearable sensor*" OR "fitness tracker*" OR smartwatch* OR "activity monitor*" OR "accelerometer-based monitor*" OR "sensor-based assessment*" OR "motion sensor*" OR "physiological monitor*" OR "biometric monitor*" OR biosensor* OR "physiological signal*" OR "biometric signal*" OR "electrocardiogram" OR ECG OR EKG OR "electromyography" OR EMG OR "electroencephalography" OR EEG OR cortisol OR "salivary cortisol" OR "stress hormone*" OR "pupillometry" OR "pupil dilation" OR "stress response marker*"
3. "mental health" OR stress OR burnout OR "post-traumatic stress" OR trauma OR "secondary traumatic stress" OR STS OR fatigue OR "moral injury" OR "critical incident stress" OR "psychological distress" OR anxiety OR depression OR "depressive symptom*" OR "anxiety symptom*" OR "emotional dysregulation" OR "affect regulation" OR "self-regulation" OR wellbeing OR "well-being" OR "well being" OR "psychological wellbeing" OR "mental wellbeing" OR "quality of life" OR QoL OR "life satisfaction" OR resilience OR coping OR "coping strategies"
4. patient* OR inpatient* OR outpatient* OR "chronic disease*" OR cancer OR diabetes OR "cardiovascular disease*" OR "neurological disorder*" OR "rehabilitation patient*" OR "hospital patient*" OR "psychiatric patient*" OR "treatment-seeking" OR "pharmacological treatment" OR "psychiatric treatment*" OR "hospitalised" OR "nursing home resident*" OR "care home resident*" OR military OR veteran* OR army OR navy OR airforce OR "armed forces" OR soldier*
5. 1, 2, 3 NOT 4 (all within title and abstract)
6. Limit to English language
7. Limit to publication year 2000–2025
8. Limit to peer reviewed
9. Limit to adults
10. Limit to human
