# Supplementary material for: Examination of Relationships Between 24-Hour Movement Behaviors and Mental Health Outcomes in Frontline Workers: Protocol for a Scoping Review
Source: JMIR Res Protoc. 2026 May 27;15:e90813. doi: 10.2196/90813 (PMC13215630; doi:10.2196/90813)
Supplement: Checklist 1 [file resprot-v15-e90813-s002.docx]

# **PRISMA-ScR Checklist**

| **PRISMA-ScR Checklist Item** | **Status** | **Notes** |
| --- | --- | --- |
| **Title**: Identify the report as a scoping review protocol | ✅ Complete | Title clearly identifies the review as a scoping review protocol. |
| **Abstract**: Structured summary | ✅ Complete | Abstract includes background, objectives, eligibility, information sources, and synthesis plan. |
| **Rationale**: Describe rationale for the review | ✅ Complete | Comprehensive justification for a scoping review approach provided. |
| **Objectives**: State the questions and objectives | ✅ Complete | Primary and secondary questions are clearly defined. |
| **Eligibility Criteria**: Define inclusion/exclusion criteria | ✅ Complete | Detailed criteria for population, concept, context, and study types are provided. |
| **Information Sources**: Describe databases and coverage dates | ✅ Complete | Multiple databases listed with date range (2000–present). |
| **Search Strategy**: Present draft search strategy | ✅ Complete | Search strategy sample for APA PsycInfo is provided in Multimedia Appendix 2. |
| **Selection of Sources**: Describe screening process | ✅ Complete | Title, abstract and full-text screening by independent reviewers using Rayyan. |
| **Data Charting Process**: Describe data extraction approach | ✅ Complete | Piloted standardised data charting form with dual reviewers. |
| **Data Items**: List variables and definitions | ✅ Complete | Full list of extracted variables provided. |
| **Critical Appraisal of Sources**: If conducted, describe methods | ✅ Not applicable | No critical appraisal planned, in line with JBI guidance for scoping reviews. |
| **Synthesis of Results**: Describe planned synthesis method | ✅ Complete | Narrative synthesis and descriptive mapping described, with stratification where appropriate. |
| **Ethics and Dissemination**: Address ethics approval and dissemination plans | ✅ Complete | Ethics exemption explained; dissemination strategy includes academic, stakeholder, and public. |
| **Funding**: Describe sources of funding and funder role | ✅ Complete | Funded by a Strathclyde Centres for Doctoral Training (SCDT) award and Industry funding from Sentinel Ltd |
